# High-Temporal-Resolution Object Detection and Tracking Using Images and Events

**DOI:** 10.3390/jimaging8080210

**Published:** 2022-07-27

**Authors:** Zaid El Shair, Samir A. Rawashdeh

**Affiliations:** Department of Electrical and Computer Engineering, University of Michigan-Dearborn, Dearborn, MI 48128, USA; srawa@umich.edu

**Keywords:** event-based vision, object detection and tracking, high-temporal-resolution tracking, frame-based vision, hybrid approach

## Abstract

Event-based vision is an emerging field of computer vision that offers unique properties, such as asynchronous visual output, high temporal resolutions, and dependence on brightness changes, to generate data. These properties can enable robust high-temporal-resolution object detection and tracking when combined with frame-based vision. In this paper, we present a hybrid, high-temporal-resolution object detection and tracking approach that combines learned and classical methods using synchronized images and event data. Off-the-shelf frame-based object detectors are used for initial object detection and classification. Then, event masks, generated per detection, are used to enable inter-frame tracking at varying temporal resolutions using the event data. Detections are associated across time using a simple, low-cost association metric. Moreover, we collect and label a traffic dataset using the hybrid sensor DAVIS 240c. This dataset is utilized for quantitative evaluation using state-of-the-art detection and tracking metrics. We provide ground truth bounding boxes and object IDs for each vehicle annotation. Further, we generate high-temporal-resolution ground truth data to analyze tracking performance at different temporal rates. Our approach shows promising results, with minimal performance deterioration at higher temporal resolutions (48–384 Hz) when compared with the baseline frame-based performance at 24 Hz.

## 1. Introduction

Object tracking is a common and well-defined task in computer vision. It entails identifying objects in a scene and tracking their locations across time. The implementations using conventional cameras have been vast and well-established for quite some time [1,2,3]. Typically, object trackers utilize an object detection mechanism applied to images, to detect and track present objects across sequential frames based on some association metrics. This results in discrete tracking outputs with rather low temporal resolution, even when the object detection performance is ideal. Such temporal resolutions might be insufficient for high-speed robotics or for applications that require higher tracking temporal resolutions.

Most conventional cameras (hereafter referred to as frame-based cameras) capture images at a relatively low fixed rate of about 30 Hz (or frames per second). Low dynamic range, motion blur, high power consumption, as well as low update rates, are among the main limitations of frame-based cameras.

On the other hand, event-based vision, which is an emerging field of computer vision, proposes a novel type of bio-inspired sensing modality that offers different physical properties that can be utilized for common computer vision tasks, including object detection and tracking. These sensors, commonly known as event cameras in the literature, capture per-pixel brightness changes at a very high temporal resolution at the level of microseconds. These brightness changes are referred to as events and are only generated whenever the brightness change of any given pixel exceeds a set threshold. An initial version of this sensor, known as the Dynamic Vision Sensor (DVS), was first introduced in 2008 by Lichtsteiner et al. [4]. 

In general, an event can be defined as:

(1) e={x, y, t, p},

where x and y denote the 2D pixel coordinates of the event, whereas t is the timestamp in microseconds of when the event was captured, and p specifies the polarity of the event, which can be either positive or negative p∈{+1,−1}, indicating a brightness increase or decrease, respectively. 

Unlike frame-based cameras, event cameras generate data asynchronously only at the pixel(s) that undergo a brightness change. These brightness changes (events), other than noise, typically imply motion or highlight changes in the scene. Moreover, event cameras offer numerous advantages compared to standard cameras, including a high dynamic range (HDR) of typically >120 dB vs. ~60 dB for standard cameras, no motion blur, low latency (microseconds), high temporal resolution (~1 µs per event), and low power consumption [5]. A more in-depth literature survey of this technology can be found in [6].

When it comes to object tracking, the limitations of frame-based cameras can affect performance. Considering their low capture rates, a rapid change in the position or motion of an object being tracked, for example, might not be detected if it occurs at a higher rate than the camera’s capture rate. The effects of this might cause undesired outcomes depending on the intended application, as tracking ends up yielding a low temporal resolution output with insufficient data for the inference of other useful characteristics, such as object kinematics (velocity and acceleration rates), or the ability to generate continuous tracking results without the use of data extrapolation techniques.

As for the other frame-based limitations, object tracking can suffer intermittent object detection performance, where objects of interest are not always successfully detected in each frame. This causes some false-negative readings (missed detections) that may result in erratic and inconsistent tracking performance, especially if other means of averaging or filtering are not applied. Theoretically, the maximum achievable tracking rate should be bounded by the camera’s synchronous capture rate, generated at discrete times, given an ideal object detection and tracking performance. Alternatively, a high framerate input source can be used to yield higher tracking resolutions. However, frame-based object detection is computationally expensive and can be very significant in this case, as inference times per frame are usually in the order of several milliseconds, at best using deep learning-based object detectors [7]. This can effectively limit real-time performance, which might be needed given the application. Moreover, consecutive frames might have minimal changes between them, creating redundant data, yet with the same computational expense per frame. This is in addition to the fact that high framerate cameras are expensive, require more memory, and consume more power [8].

Nonetheless, event cameras suffer from limitations as well, one of which is the lack of intensity information that regular cameras provide, which causes object classification to be challenging. Although it was shown that intensity images can be reconstructed from events [9], noise and other issues can cause artifacts in the reconstruction. This is evident in scenes with limited changes generated by a camera without any ego-motion applied, in which a significant proportion of the events generated are due to noise. Ego-motion is defined as the 3D motion of a camera relative to the environment [10]. Ego-motion applied on an event camera acts as a trigger that generates events at the edges of the objects within the camera’s field of view due to the brightness changes prominent around edge-like features. Accordingly, to achieve more robust detection and tracking, a combined approach would be advantageous.

**Figure 1:**
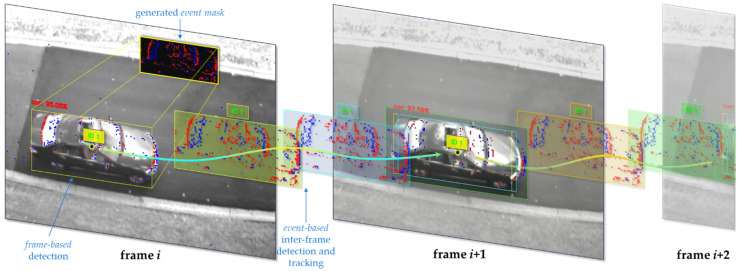
A conceptual diagram of our high-temporal-resolution object detection and tracking approach using images and event data. The figure shows three sequential grayscale image frames across time, with events (red and blue dots) overlayed on top, representing their sparse and asynchronous nature. An event mask is extracted whenever an object is detected in a given image, which is then used for inter-frame detection and tracking using events until a new image is captured and the process is repeated.

In this paper, our main contributions can be described as follows:

1. We present and evaluate a novel hybrid approach to utilize some of the advantages of both types of sensing modalities (frame-based and event-based vision) to produce higher tracking temporal resolutions. Frame-based vision is used for detecting and classifying objects in a scene (learned approach), whereas event-based vision’s asynchronous and high temporal resolution is used for inter-frame tracking by using *event masks* extracted from the event stream guided by the frame-based detection position (classical approach). *Euclidean* distance-based object association is used, as the data generated is assumed to be continuous whenever an object is moving, to evaluate the feasibility of higher temporal resolution tracking. Our approach is demonstrated in Figure 1.
2. We collected and manually labeled several hours of synchronized image and event data using Dynamic and Active-Pixel Vision Sensor (DAVIS) 240c (Zurich, Switzerland) [5], which combines a grayscale camera, known as Active Pixel Sensor (APS), as well as the event-based sensor DVS, using the same pixel array. Our labeled data provides both the true 2D bounding boxes for all vehicles in the scene for any image, as well as their corresponding object IDs, which are used for object tracking evaluation. This dataset is publicly available.
3. To generate matching high-temporal-resolution tracking data for our evaluations, we temporally interpolate our ground truth data multiple times to yield true rates beyond the base framerate of the APS, which is 24 Hz.
4. We assess our approach’s performance using state-of-the-art object detection and tracking metrics, at temporal resolutions of 48, 96, 192, and 384 Hz.

The remainder of this paper is organized as follows. In Section 2, the related work is surveyed. Section 3 breaks down our hybrid tracking approach. Section 4 describes the experimental setup used to evaluate our approach, including the dataset, experimental configuration, and metrics used. Section 5 presents and discusses the results, and Section 6 concludes our work.

## 2. Related Work

### 2.1. Frame-Based Object Tracking

Frame-based multi-object tracking has been well-established in the literature for quite some time. Most works currently utilize direct methods, specifically *tracking-by-detection*, using optimized object detectors, while focusing on the data association aspect of object tracking [11,12,13,14,15]. 

Recent state-of-the-art trackers, such as DeepSORT [12] and SOTMOT [13], propose different association methods that are performed in an *online* manner and constrained by a trade-off between accuracy and latency. DeepSORT [12], for instance, incorporates motion information based on a recursive Kalman filter [16] and appearance information generated by a pre-trained convolutional neural network (CNN), using *Mahalanobis* distance, to perform data association on a frame-by-frame basis. Frame-based detections are generated using a fine-tuned FasterRCNN [17]. Meanwhile, SOTMOT [13] employs a one-shot framework based on a modified DLA [18] backbone with multiple parallel branches to perform object detection and data association simultaneously. 

*Global* methods, also known as *batch* methods, exist as well [14,15]. However, they are not considered in this paper due to their limited utility in robotics operating in real time, as they function in an *offline* manner. Thus, they require full knowledge of all present and future data for object detection and tracking. Further, it is common for global trackers (and some online ones) to use linear interpolation to cover the gaps in the trajectories of the objects being tracked. On the other hand, some trackers (such as Deep SORT [12]) incorporate motion information to improve data association and mitigate missing detections, using predictions generated by a Kalman filter [16]. Finally, most of these implementations are usually evaluated and compared using common frame-based multi-object tracking benchmarks, such as MOT20 [19], which contains only image frames (no event data).

In our work, we use Euclidean distance [20] as the data association metric. Euclidean distance can be defined as the length of a line connecting any two points. This metric is sufficient for our work, given the modest complexity of the dataset used and the expected continuous nature of the object detection resulting from the added use of event data. Furthermore, in our work, the frame-based-only approach to object tracking is only used as a baseline (at 24 frames per second) to compare with the tracking results of the higher temporal resolutions (48 Hz and above) that use both modalities. Thus, it is irrelevant to include other frame-based approaches in our evaluation. Finally, to constrain the scope of this work, we do not investigate the application of interpolation techniques to fill any gaps in the generated tracking trajectories.

### 2.2. Event-Based Object Tracking

In contrast with frame-based object tracking, event-based object tracking is still in its early stages. In the literature, event-based feature tracking has been the focus of the research community and significant progress has been made. It entails using event data to extract features of different types (e.g., corners) and track them through time [21,22,23]. As for event-based object detection and tracking, most works have been application-specific, with few similarities overall [24,25,26,27,28,29,30,31,32,33,34]. We categorize these works as either *event-based* or *combined* (i.e., using images and events) approaches. 

A common approach to event-based object tracking is using clustering methods [25,26,31,34]. Clustering is an intuitive approach for event-based object tracking whenever there is no ego-motion applied to the camera, thus assuming that events are mainly generated around the moving objects. Therefore, these clusters can track these objects with decent performance. Nevertheless, clustering is less robust against occlusion and can lead to more object ID switching between the objects being tracked.

As for the other, non-clustering, event-based tracking methods, Mitrokhin et al. [27] proposed a motion compensation model that enables the detection of objects in a scene by finding inconsistencies in the resulting model and then tracking them using a Kalman filter. They tested their approach on a dataset collected on a moving platform comprising several sequences of varying lighting conditions. The objects were labeled at the time instances of the captured RGB frames. Finally, they evaluated their tracking performance based on a success rate of the percentage of objects detected with at least 50% overlap. 

Chen et al. [29] proposed an asynchronous tracking-by-detection method for object tracking based on bounding boxes which involved combining events and converting them into frames. Afterward, they used the generated frames with their proposed tracking method and directly compared them with other frame-based approaches. The number of frames generated is dynamic, based on the sum of events captured due to the motion of the objects in the scene. Objects are detected using a contour-based detector, then tracked using an Intersection over Union (IoU) measure for data association. Finally, they used the same dataset provided in [27] along with average precision (AP) and average robustness (AR) metrics for evaluation.

Ramesh et al. [28,35] presented an object tracking method using a local sliding window technique for reliable tracking. Objects are initially detected using a global sliding window to find regions of interest (ROIs) which is only used during the initialization of an object or when the tracking fails to enable real-time performance. Finally, overlap success and center location error metrics were used for quantitative evaluation on a short indoor data sequence [36].

As for the combined approaches using events and frames, the work by Liu et al. [24] proposed to utilize the event stream to generate ROIs using cluster-based methods which are then classified by a CNN as either foreground or background. Finally, a particle filter is used to estimate the target’s location using the extracted ROIs. This work was mainly meant for detecting and tracking a single object (representing a prey robot); therefore, positional accuracy was used as the evaluation metric.

Zhang et al. [32] similarly presented a multi-modal approach to achieve single object tracking. They evaluated success and precision rates on a large-scale dataset annotated at different frequencies, for both vision domains, using a motion capture system. Meanwhile, Zhao et al. [33] proposed an object detection method based on color which then tracks a single object using a kernel correlation filter applied to the event data and estimates the distance to the object, while mean average precision (mAP) is used to assess the detection performance.

Overall, we noticed that most works in the literature focused on object tracking from a detection perspective, meaning that they only estimated the overall detection and overlap success rates for all objects available. None seems to have evaluated data association performance, which is the common practice in the frame-based domain. This can be attributed to the scarcity of event-based datasets as well as the limitations of the publicly available ones, as most authors emphasized single object tracking and thus did not include ground truth object ID data per annotation. Object IDs are required by the most popular object tracking metrics [3,19,37] for evaluating data association performance. In contrast, we provide a fully labeled traffic dataset with bounding boxes and object IDs for objects of vehicle type. Additionally, to the best of our knowledge, none of the works have explored the use of event data for higher temporal resolution object tracking than the base framerate of a given frame-based camera. Meanwhile, we achieve this here by generating several higher-temporal resolution ground truth data for the acquired sequences, at various rates. These labeled trajectories are then utilized in the evaluation of different approaches for event-based inter-frame frame tracking, using well-defined object tracking metrics [3,19,37]. Accordingly, we assess the feasibility of high-temporal-resolution tracking using a hybrid approach.

## 3. Hybrid Object Tracking

In this section, we break down the design of our hybrid approach.

### 3.1. Frame-Based Object Detection

Given temporally synchronized streams of images (frames) and event data, we start with the image stream. A vital first step for tracking objects across time is to detect them when they first appear and in every subsequent frame. As mentioned before, classification using event data alone is challenging; therefore, our approach uses the image frames to detect and classify objects wherever they appear in the scene, then tracks them between frames using event data. 

**Figure 2:**
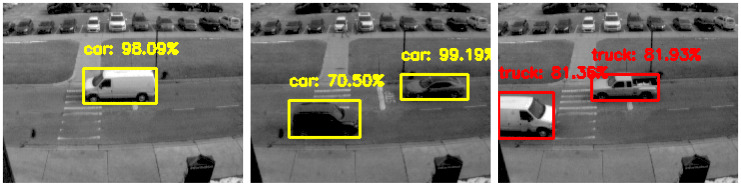
Object detection on some sample images (**left**, **center**, and **right**) of our collected data for one of the scenes. The object detector used in this figure is YOLOv3. In this scene, static objects, such as the parked vehicles in the top half of the scene, are disregarded.

To achieve reliable object detection, we utilize two well-known, pre-trained, deep-learning-based object detectors, namely, YOLOv3 [38] and SSD [39], to perform frame-based object detection. These models are used to detect objects in every new image frame, as shown in Figure 2, initializing the objects to be tracked and feeding into the Euclidean-based object tracker, described in Section 3.3. The frame-based object detectors can be replaced by other frame-based detectors as needed based on the desired minimum accuracy and maximum latency requirements. In our work, we use a detection confidence threshold of 50% and a non-maximum suppression threshold of 50% as well for both object detectors used. This process is repeated whenever we read a new image frame.

### 3.2. Event-Based Object Detection

#### 3.2.1. Combining Image and Event Streams Using Window Frames

**Figure 3:**
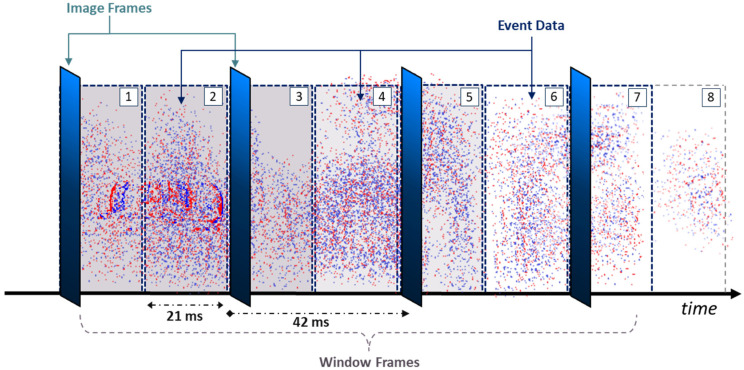
Visualization of a synchronized stream of image frames and event data over time. In this example, the image frames are captured at around every 42 ms (at a rate of 24 Hz), whereas the window frame size is set to a temporal resolution of 21 ms (tracking rate of 48 Hz). A window frame encapsulates any image frames and event data available in that specified time frame. A total of 8 window frames are demonstrated in this figure as indicated by their number.

To make use of an asynchronous event stream, an event-representation method is required. In our work, we accumulate events for a certain interval and incorporate them into a *window frame*, along with any available image frames. For our application of high-temporal-resolution tracking, the desired tracking rate k must be initially set. k defines the tracking rate our system would utilize to accumulate and parse event data. For example, given that the frames are captured at a rate of 24 Hz, a k value of 48 Hz would indicate that a window frame is collected every 21 ms. The *window frame size* refers to the duration of the time that the system will read and accumulate synchronized images and event data per window frame. As stated earlier, DAVIS 240c has a frame-based capture rate of 24 Hz; therefore, a new image frame is read around every 42 ms. Thus, using a k value of 48 Hz, every other window frame will contain an image frame (captured by the APS) as well as all the events generated throughout that time (captured by the DVS). This is demonstrated in Figure 3. In this paper, we experiment with multiple k values, including 24, 48, 96, 192, and 384 Hz, which correspond to window frame sizes of around 42, 21, 10, 5, and 3 ms, respectively.

Accordingly, whenever a window frame containing an image is read, frame-based object detectors output a list of 2D bounding boxes with corresponding object classes for each, as described in Section 3.1. Whenever these detections are fed into the object tracker, we generate an event mask per object detected. These event masks are used to accurately detect and localize the identified objects, using the event data, in the subsequent window frames containing events only (assuming that k is higher than the APS base frame rate). Using the prior example (k= 48 Hz), the first window frame would contain an image as well as events, whereas the second would only contain events. Similarly, the third window frame would contain both, while the fourth would contain only events, and so on, as shown in Figure 3.

Furthermore, the window frame can either take discrete time steps or use a moving window instead. A discrete step would mean that the window frame would move $\frac{1}{k}$ ms forward for every new frame, as shown in Figure 3. Meanwhile, a moving window would incorporate a longer duration of event history for every window frame; thus, some events would be included in multiple consecutive ones. For example, when setting the event-history duration as 50 ms and the tracking rate as 48 Hz, the window frame would read the last 50 ms of event data at any time instant $t_{i}$ (instead of just 21 ms in the case of discrete time steps), yet it would still move 21 ms forward when loading a new window frame. In general, the window frame would include all of the events available within the time interval $\left\{t\in {\mathbb{R}}_{+}\;|\;t_{i}-50\;ms\;\leq t\;\leq \;t_{i}\right\}$ at a given time instant $t_{i}$. Incorporating a longer temporal history of events can produce higher tracking accuracy, especially at greater tracking rates or resolutions, where larger numbers of event data are accumulated compared to when using a discrete-step window frame. The effects of both parameters, as well as temporally weighting the events, are evaluated later on in this paper.

#### 3.2.2. Event Mask Extraction

**Figure 4:**
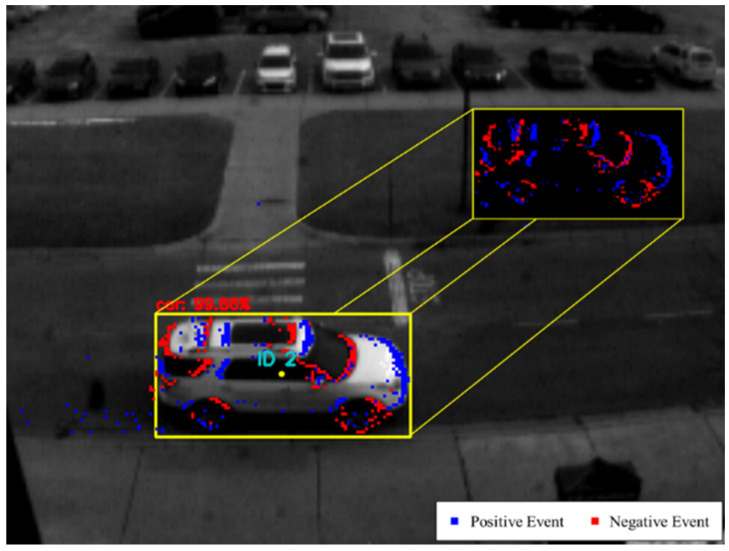
The figure demonstrates when an event mask is generated by accumulating the events located within the bounding box, as shown in the top right corner. In this frame, a white SUV is detected, as highlighted by the yellow bounding box (using the frame-based object detector SSD [39]), with 99% confidence. The tracking rate used here is 48 Hz, meaning that the window frame’s size is 21 ms, and only the events captured during this interval are displayed.

As for the event masks, they can be either *event-based* or *edge-based*. Event-based masks are produced by extracting all the accumulated events (available in the most recent window frame) that are located within the bounding box of each object detected in the image, as shown in Figure 4. Due to the sparse nature of event data, the event-based masks are stored as a sparse matrix of +1 and −1 integers, representing the mask’s positive and negative events, respectively. Additionally, only the most recent event per pixel is used in the event-based mask’s sparse matrix. Moreover, if a discrete-step window frame is used, the event mask appends the events found in the next window frame after the object is tracked to improve the tracking robustness in subsequent window frames containing events only. However, this approach assumes that an object is correctly tracked using the event data. Otherwise, if a moving window with a significant amount of event history is used, the event mask is only generated when detecting an object in a given image frame and used without alteration in the subsequent window frames of event data.

**Figure 5:**
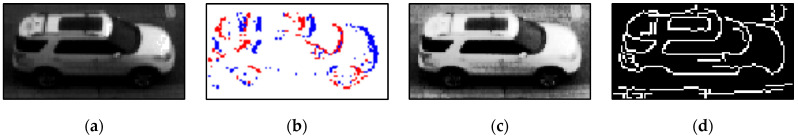
Visualization of (**a**) the actual grayscale image crop based on the bounding box of the detected object; (**b**) event-based mask created from the accumulated events in the current window frame used in event-based tracking; (**c**) a histogram-equalized version of the crop; and (**d**) the generated edge-based mask used for event-based tracking as well.

On the other hand, edge-based masks are generated using the image’s bounding box crop, generated by the frame-based object detector. Given that events are typically generated around the edges of an object whenever there is motion, an edge-based mask can be useful for event-based tracking. To generate an event-based mask, the bounding box crop is initially converted to grayscale (if an RGB image is used), then it is equalized based on its histogram to mitigate low-contrast crops that are either too dark or bright to be able to generate accurate edges. Afterward, an edge-based mask is generated using the *Canny Edge Detection* algorithm (developed by Canny, J. [40]) which is then thresholded to create a binary version of zeros and ones (representing the object’s contour). Finally, it is stored in a sparse matrix that represents the event mask of the object. These steps are demonstrated in Figure 5. Note that when an edge-based mask is used, the event polarities are no longer utilized. Instead, only the presence of an event at a given pixel is considered.

The motivation behind the edge-based approach is that events are mainly generated at the edges of the objects, as edges represent a sharp intensity change in a given local patch of an image. This way, an edge map would be more robust with respect to tracking an object moving in any direction, whereas, for an event-based mask, events are generated in the direction of motion; therefore, if an object suddenly moves perpendicularly to its prior direction of motion (e.g., vertically instead of horizontally), tracking might momentarily fail until sufficient events are captured and accumulated due to the vertical motion. We can notice this effect on the event-based mask in Figure 5b. The edges around the top and the bottom of the vehicle have almost no events in contrast to the edge-based mask in Figure 5d. Nevertheless, the edges of the background are also incorporated into the mask, which might affect the tracking’s accuracy and precision. Additionally, the edge-based mask can be affected by poor image conditions, specifically when there is over- or underexposure in the scene.

#### 3.2.3. Inter-Frame Object Detection Using Event Data

Once the initial window frame containing an image frame is read, the next window frame is loaded. Assuming a k value of 48 Hz, the second window frame would contain event data only (as demonstrated earlier in Figure 3). Therefore, the next step would be to perform event-based object detection and tracking, using the extracted event mask of each object detected in the prior window frame’s image. Similar to [28], a *search region* is used to track an object locally using the available events.

**Figure 6:**
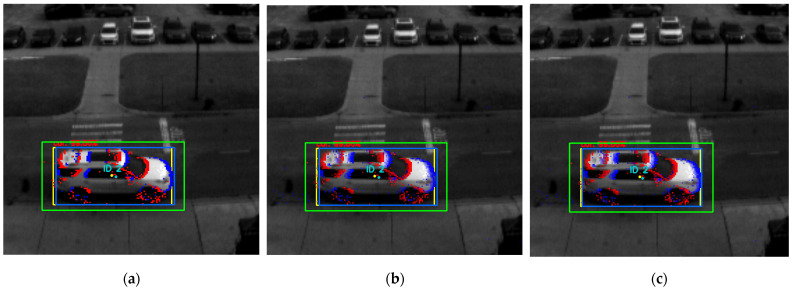
Inter-frame tracking output at 48 Hz in three different modes: (**a**) event-based mask with a discrete-step moving window with no temporal weighting; (**b**) event-based mask with temporally weighted events in a 50 ms moving window frame; (**c**) edge-based mask with temporally weighted events in a 50 ms moving window frame. The inter-frame object position is highlighted by the light-blue bounding box (cyan dot represents its centroid), whereas the yellow bounding box and dot represent the object’s position and centroid in the latest image frame, respectively.

**Figure 7:**
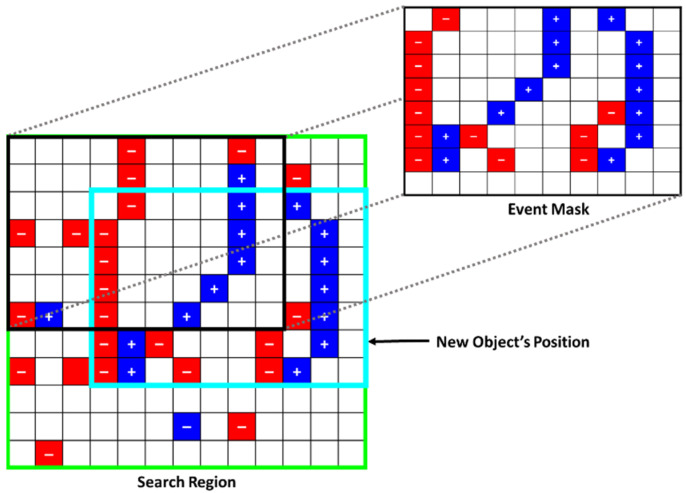
Demonstration of the sparse matrix multiplication between the event mask and a sliding section of the search region. This process is used to find the highest correlating position of the object by summing the result of each multiplication, similar to a typical image convolution using a kernel. Based on the results of the sliding window mechanism, the new object’s location is set by selecting the highest correlating position (highlighted by the cyan rectangle in this example).

Based on the set parameters, the event-based inter-frame object detection and tracking is performed as follows:

1. Create a search region positioned around the center of each of the objects being currently tracked (detected in the latest image). The search region is set 20% larger than the frame-based detection’s height and width. Thus, around a 44% larger bounding box size is used in our case (represented by the green bounding boxes in Figure 6). This value can be set according to the nature of the objects (expected velocities, etc.). Larger search regions can be used, however, at higher computational costs. Moreover, we add padding to the search region when an object is at the edge of the frame and is exiting the scene, to return a more accurate object position.
2. Extract all the events (available in the current window frame) located within the search region.
3. For every possible event mask and search region intersection combination:
  - Using a sliding window mechanism, create a sparse matrix of the subset of the search region. These events are encoded either *spatially* or *spatiotemporally*.
  - Perform a cross-correlation between the mask and every search region’s subset, as demonstrated in Figure 7. This process is mainly a two-dimensional sliding-window matrix multiplication between the event mask and each subset of the search region (starting at the top left corner of the search region). The sum of all the cells, resulting from every matrix multiplication combination, is stored in the corresponding entry of the cost matrix C. The cost matrix C is of size m rows by n columns, which are defined as:
    (2) $$m=H_{sr}-H_{em}$$
    (3) $$n=W_{sr}-W_{em}$$
    where $H_{sr}$ and $W_{sr\;}$ are the search region’s height and width, while $H_{em}$ and $W_{em}$ are the event mask’s height and width, respectively.

4. Based on highest $C_{i,j}$ entry value, use the best correlating box as the object’s inter-frame position. Figure 7 shows the best tracking result of this maximum correlation step highlighted in the cyan bounding box, which is the best fit for event-based tracking for the current window frame. Similarly, this is demonstrated in Figure 6 by the light-blue bounding boxes. A minimum threshold is typically applied so that the system will only update each object’s position if the $C_{i,j}$ value is above a set threshold. This is typically done to avoid updating the object’s position based on noise, thus limiting the number of false positives.
5. If successfully detected, update the object’s position using the object tracker described in Section 3.3. If a discrete-step window frame is used, update the object’s event mask by aggregating it with the new event data available within the updated position, assuming the object is correctly detected and that the new events will line up correctly with the previous ones. This step typically improves the tracking robustness, particularly when tracking at very high rates (e.g., >200 Hz), at which fewer events are captured. Otherwise, if a moving window frame is used, the event mask would only update once a new image frame is read (given that the event-history length is sufficient).
6. Finally, load the next window frame and repeat the same process according to if it contains an image or just event data.

**Figure 8:**
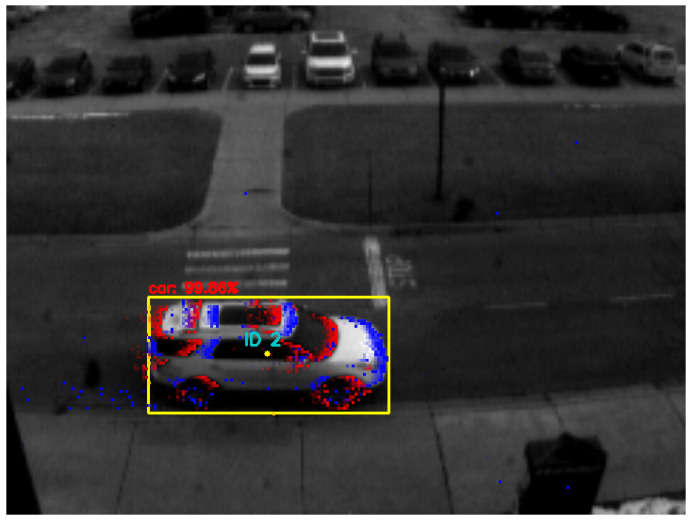
The figure demonstrates an image with temporally weighted events (visualized by the transparency effect) overlayed on top. Faded blue and red dots resemble older positive and negative events, respectively. This scene represents the same time instance as the one shown in Figure 4 at a tracking rate of 48 Hz, though with an extended 50 ms of event data history compared to 21 ms.

Note that when creating the search region (step 3a) to find the object’s inter-frame position, we encode the events either spatially or both spatially and temporally. Spatial encoding refers to incorporating the events’ x and y coordinates in the tracking process (which is the base case throughout the paper), whereas temporal encoding incorporates their capture time t as well. Temporal encoding is accomplished by weighting the events either equally or temporally. Equal weighting gives all events the same significance, meaning that all events have the same impact on the estimated position of the object. Meanwhile, temporal weighting gives more weight to the most recent events and less weight to the older events. This is visualized in Figure 8.

To weight the events *temporally*, we use the following equation for each event:

(4) $$w_{e_{i\;}}=\frac{p_{e_{i}}*\left(t_{e_{i}}-t_{w_{j}0}\right)}{\Delta t_{w_{j}}}$$

where $w_{e_{i}}$ is the given weight of the event $e_{i}$ at a specified pixel position; $p_{e_{i}}$ and $t_{e_{i}}$ are the polarity and the timestamp of the event $e_{i}$, respectively; while $t_{w_{j}0}$ and $\Delta t_{w_{j}}$ are the window frame j’s start time and size (in the same timestamp unit). As described in Section 3.2.1, the window frame size would be equal to either $\frac{1}{k}$ ms, if a discrete-step window is used, or a specified duration (longer than $\frac{1}{k}$), if a moving window is used with an extended event history. The resulting weights $w_{e}$ are appended to the search region’s sparse matrix (using the most recent event available at every pixel coordinate), then used in finding the best object position estimate. In contrast, when the events are weighted equally, the weight $w_{e_{i}}$ of each event is simply set equal to their defined polarities $p_{e_{i}}$. Moreover, the polarity $p_{e_{i}}$ of any event is set as 1 when using an edge-based event mask to track the objects.

### 3.3. Euclidean-Based Object Tracker

As for the object tracker, we use a simple centroid-based (detections’ center x and y coordinates) object tracking algorithm using Euclidean distance [41] as the object association cost across consecutive window frames. Euclidean distance is a metric that is used to find the optimal assignments to be able to track objects across subsequent frames at any given point with a low computational cost. Moreover, it is appropriate for our application given the continuous nature of the event data and the presumed object detection data, as the centroid of any moving object should be the one closest to its prior center, given that it was successfully detected. The centroid-based tracking algorithm used is based on the work of Adrian Rosebrock [41].

Even though the inter-frame event-based detection (described in Section 3.2.3) fundamentally tracks the objects and estimates their new positions, the detection results are fed into the object tracker to confirm the object assignments. The object tracker uses these detections to either: register new objects with a unique ID, update the positions of the current ones being tracked, or possibly remove the objects that were not successfully matched for n subsequent window frames. Overall, more sophisticated association metrics can be used; however, this work mainly focuses on presenting a novel method to leverage the event data to enable higher-temporal resolution tracking and analyze its feasibility. Thus, the object tracker can be replaced by other tracking-by-detection methods in future studies as desired.

**Figure 9:**
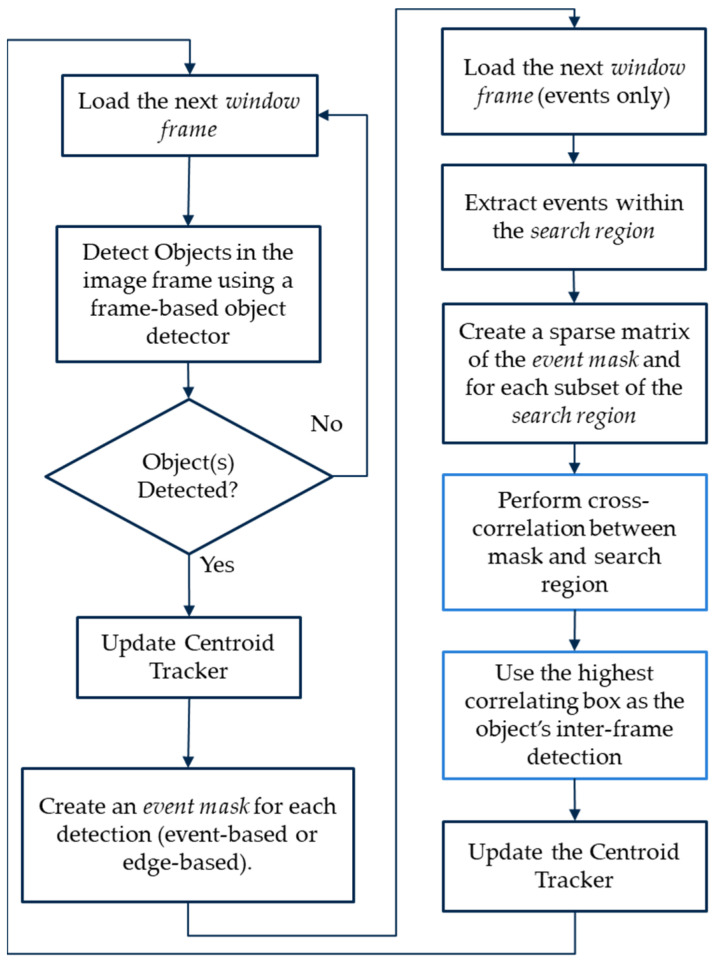
Summary flowchart of the overall hybrid object detection and tracking process. The branch on the right would repeat for every consecutive window frame that only contains events given prior frame-based object detections until a new image is read. The window frame size is set before this process starts.

Lastly, we summarize our overall object detection and tracking approach in Figure 9. 

## 4. Experimental Setup

In this section, we describe the dataset that was collected and labeled and utilized in the evaluation of our approach, then we define the object detection and tracking evaluation metrics used, and, finally, we overview the different tracking configurations applied in our experiment.

### 4.1. Dataset Description

To evaluate our approach’s tracking performance, we used the DAVIS 240c [5], which combines a frame-based sensor APS, and an event-based sensor DVS, to collect our evaluation dataset. The DAVIS 240c uses the same pixel array for both sensor types with a resolution of 240 × 180 pixels. The APS captures synchronous intensity (monochrome) images at a fixed rate of ~24 Hz. Meanwhile, the DVS captures asynchronous events with a temporal resolution of 1 µs.

Using the DAVIS 240c and the ROS DVS package developed by Robotics and Perception Group [4,5,42] to record the data, we collected several hours of spatially and temporally synchronized images and events at two different scenes, referred to as scenes A and B. Scene A was demonstrated earlier in the figures of Section 3. In addition, we note that the proximity of the objects to the event camera in scene B were lower than in A; therefore, the objects detected in the scene were larger in size relative to the frame. Snapshots of scenes A and B were shown in Figure 2 and Figure 1, respectively.

As for the setup, the event camera was placed on the edge of a building while pointing downwards at the street, representing an infrastructure camera setting. The camera was static (no ego-motion was applied); therefore, the events captured would be only due to an object’s motion or due to noise. In our experiment, we mainly captured data sequences of moving vehicles of different types (sedans, trucks, etc.). Some data of pedestrians passing by were also collected but they were not included in this study due to the relatively slow movements of the pedestrians and their proximity to the camera, which made the object detection challenging and intermittent. Further, we split each scene’s recorded data into ~30 short sequences that mostly contained images and events of objects present in the scene while trimming the other intervals that did not contain any. We also note that the vehicles that passed by in the scene did so at varying speeds and accelerations, some reaching a full stop at several instances, thus making event-based detection and tracking more challenging.

In order to quantitatively evaluate our hybrid-based object detection and tracking approach, we manually labelled APS-generated intensity images. Our labeled data provided both the true 2D bounding boxes for all vehicles in the scene present in any image, as well as their corresponding object IDs, which are required for proper object tracking evaluation. Accordingly, after splitting the data into more compact sequences, scene A contained 32 sequences, with 9274 images and 6828 annotations, while scene B contained 31 sequences, with 3485 images and 3063 annotations, totaling 9891 vehicle annotations. The difference between the number of images and annotations was due to the frames that did not contain any objects. 

As for the high-temporal-resolution tracking experiment, we needed matching tracking ground truth data for our evaluations. Therefore, we temporally interpolated our ground truth data, based on a constant acceleration model, to increase the temporal resolution of our ground truth data and produce the estimated true tracking rates beyond the 24 Hz base framerate of the APS. This was done by taking an object’s annotations at 24 Hz, then finding the interpolated bounding box positive between every two consecutive labels, while maintaining the same object ID. The first interpolation yielded the ground truth tracking data for 48 Hz; thus, we repeated the same process multiple times to generate the ground truth tracking data for temporal resolutions of 96, 192, and 384 Hz as well. Overall, this intuitive method provided us with the ground truth labels for inter-frame tracking using event data, given that directly labeling events is a very challenging task, especially at time instances with very few events resembling shapes recognizable by a human.

### 4.2. Evaluation Metrics

Many evaluation metrics are available to assess detection and tracking performance. In our experiment, we used the novel *Higher Order Tracking Accuracy* (HOTA) metric, developed by Luiten et al. [37], that is used to evaluate multi-object tracking performance. HOTA is particularly useful in assessing the performance of object trackers, as it analyzes the accuracy of the detection, association, and localization of the objects individually and combines them within the same metric. To calculate the final HOTA score, the Intersection over Unions (IoUs) of localization, detection, and association are calculated. IoU is simply defined as the ratio of the overlap of two detections over their total covered area. The two detections used in the IoU calculation are typically the predicted and the true ground truth detections. 

As defined by the authors, the foundation of the overall HOTA metric can be described as follows:

- *Localization Accuracy* (LocA) is the average of all localization IoUs between all possible pairs of matching predicted and true detections of the dataset. Localization refers to the spatial alignment of the predictions compared to the ground truth detections.
- *Detection Accuracy* (DetA), similar to LocA, measures alignment between the set of all predicted and ground truth detections. However, it incorporates a defined IoU threshold α to identify which predicted and true detections intersect to find the matching pairs, known as True Positives (TPs). False Positives (FPs) are the predicted detections that do not match, while False Negatives (FNs) are the ground truth detections that do not match. Accordingly, DetA is calculated by dividing the total count of TP over the summation of the count of TPs, FPs, and FNs.
- *Association Accuracy* (AssA) measures how well a tracker associates detections over time using all object IDs, i.e., assesses the whole track of each ground truth object ID using IoUs. For each track, the IoU is calculated by dividing the number of TP matches between the two tracks, divided by the summation of TP, FN, and FP matches between them as well. Ultimately, the AssA is calculated by finding the association IOU over all matching predicted and ground truth detections.
- The final HOTA value is then generated, using a range of IoU threshold α values to provide one compact value that incorporates the three different components. This value is used to assess the overall object tracking performance for a specified configuration.

Furthermore, we note that HOTA(0), LocA(0), and HOTA-LocA(0) refer to the same metrics discussed above, though at the lowest α threshold value; thus, localization accuracy does not affect the results. Additionally, DetRe and DetPr refer to the detection recall and precision performance, respectively, whereas AssRe and AssPr refer to the association recall and precision. The recall and precision values can be used to calculate the accuracy values (for both detection and association). Additional details about these metrics can be found in [37].

In addition to the HOTA metrics, we used a subset of the CLEAR MOT [19,43] metrics, including:

- *Mostly Tracked* (MT), which is the percentage of ground truth trajectories that are covered by tracker output for more than 80% of their length;
- *Mostly Lost* (ML), which is the percentage of ground truth trajectories that are covered by tracker output for less than 20% of their length;
- *Partially tracked* (PT), which is the total number of unique ground truth trajectories minus the summation of MT and ML;
- *ID-Switches* (IDSW), which is the number of ID switches or the number of times a tracked trajectory changed its ground truth one;
- *Fragmentations* (FRAG), which is the number of times the ground truth trajectory was interrupted or untracked, before resuming later.

We note that, according to the authors of these metrics, ID switches are irrelevant when measuring MT, ML, and PT. Therefore, they mostly focus on detection performance for the overall trajectory of each ground truth object, without considering association accuracy. This can provide some insight into how well an inter-frame event-based object detection system performs.

### 4.3. Experimental Parameters and Configurations

To compare and contrast the results of different detection and tracking settings, we evaluated our approach using two frame-based object detectors with three different tracking modes (of varying parameters) for event-based inter-frame object detection and tracking.

The deep-learning, frame-based object detectors used in our evaluation were YOLOv3 [38] and SSD [39]. Both of these pre-trained models provide real-time performance with great accuracy. SSD is more accurate but has higher latency when compared to YOLOv3. Both object detectors were used as is, with the original weights and without any further fine-tuning or training. Moreover, as mentioned earlier, we set the confidence and the non-maximal suppression thresholds to 50%. Lastly, we only used the ‘vehicle’ object class, including its different forms (car, truck, bus, etc.), while filtering out the other class types in our evaluation.

As for the inter-frame tracking, applied at higher temporal resolutions above the base rate (24 Hz), we used three modes of different inter-frame tracking parameter combinations:

1. Event-based mask with discrete-step moving window frame with no temporal weighting;
2. Event-based mask with 50 ms moving window frame and temporally weighted events;
3. Edge-based mask with 50 ms moving window frame and temporally weighted events.

These settings were based on the design details presented in Section 3 and are shown in Figure 6a–c. 

To summarize, we evaluated these three different modes with both frame-based object detectors and at the temporal resolutions of 48, 96, 192, and 384 Hz. As for the 24 Hz rate, we only used the frame-based object detectors, given that it matches the base capture rate of the APS, the results of which were used to set a baseline for the other tracking results and to analyze the feasibility and consistency of incorporating the event data as well to generate high-temporal-resolution tracking results. 

Additionally, we formatted our ground truth data for the different temporal resolutions and the resulting tracker outputs in the *MOTChallenge* [3] format, then generated the results using *TrackEval* [44].

## 5. Results and Discussion

**Figure 10:**
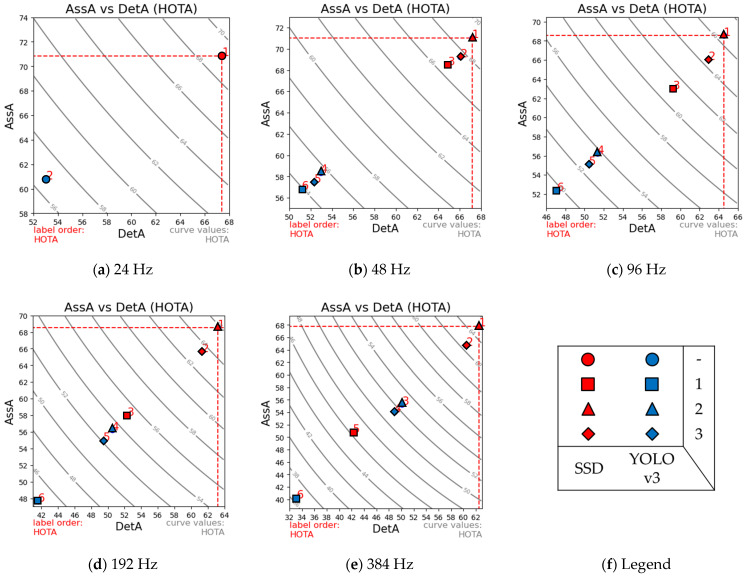
Comparison between the results of the different tracking configurations for various temporal resolutions. AssA is plotted against DetA with the resulting HOTA values marked for tracker configuration, for temporal resolutions of (**a**–**e**) 24 Hz–384 Hz. The legend (**f**) defines the symbols used according to the object detector used and tracking mode. Results show a linear correlation between the AssA and DetA, with Mode 2′s approach outperforming the other configurations for either object detector.

Based on the detection and tracking settings specified in Section 4, we obtained the results presented in Table 1 and Table 2, using the frame-based object detectors SSD and YOLOv3, respectively. Moreover, AssA values are plotted against DetA for each temporal resolution (with the resulting HOTA values) in Figure 10.

Starting with the baseline frame-based tracking results, at the base image capture rate of 24 Hz, we obtained final HOTA scores of 69 and 56.6 for SSD and YOLOv3, respectively. This was expected given that SSD is a more accurate object detector, as is highlighted by its DetA of 67.4 compared with 53.0 for YOLOv3. These values were used as the baseline values to compare our three different event-based inter-frame object tracking approaches at various temporal resolutions.

Applying the approach specified by Mode 1, which used event-based masks without history or temporal weighting, we noticed that the outcomes of most HOTA metrics significantly deteriorated with higher temporal resolutions. This is the result of a lower number of events being available to track with smaller window frame lengths. A tracking rate such as 384 Hz has a temporal interval of only 2.6 ms.

On the other hand, Mode 2, which also used an event-based mask but with a temporally weighted event history of 50 ms, consistently yielded the best performance when using either frame-based object detector. Mode 3, which used an edge-based mask instead, slightly underperformed Mode 2 but provided similar consistency. 

Overall, the approaches represented by Modes 2 and 3 proved that high-temporal-resolution tracking is possible by incorporating event data without any significant impact on performance. In Mode 2′s configuration, the HOTA values deteriorated slightly, declining from 69.0 and 56.6 (using SSD and YOLOv3 at 24 Hz) to 65.0 and 52.5. This translates to a relative performance decrease of just 5.8% and 7.24%, for SSD and YOLOv3, respectively.

Similarly, Table 3 shows the results of the selected CLEAR MOT metrics for every tracking configuration. Consistent with the previous results, Mode 2′s configuration show very minimal deterioration in tracking performance. As for the SSD-based configuration, the baseline tracking of 24 Hz had an MT of 40 and a PT of 45 with no ML objects, which was minimally affected by the higher temporal resolutions, as shown in the results for the highest rate of 384 Hz with MT, PT, and ML of 37, 46 and 2, respectively. Additionally, the YOLOv3-based configuration had MT, PT, and ML baseline tracking results of 27, 52, and 6, respectively, which insignificantly declined at the tracking resolution of 384 Hz, with only two fewer objects MT that became ML instead. Similarly, Mode 3′s configuration was a close second at varying tracking resolutions. Meanwhile, Mode 1′s configuration performed progressively worse with higher temporal results. We note that there are a total of 85 unique object trajectories in the whole dataset, as shown in Table A1. Therefore, MT, PT, and ML always add up to a total of 85. As expected, IDSW got marginally worse with increasing rates for each of the three modes, whereas FRAG suddenly increased at the temporal resolution of 96 Hz, then stabilized, except for Mode 1, which continued to worsen at increasing rates. The total number of ground truth detections for each rate is also provided in Table A1 for reference.

In general, the results show that temporal weighting of events is vital when using event-based data. Temporal information is a valuable component of asynchronous events which synchronous, fixed-rate, images lack. Our first approach, represented by Mode 1, confirms this hypothesis, where the tracking performance was significantly affected with increasing temporal resolutions, regardless of the frame-based object detector used. As for the third approach, used in Mode 3, edge-based masks were heavily dependent on the captured image quality. Given the limitations of frame-based cameras, this constrains the performance of event-based vision in challenging scenes, making the system less robust given its low dynamic range and capture rates. In our evaluation, event-based masks proved to be more robust, with lower computational costs. 

## 6. Conclusions

In this work, we have presented a novel way of using frame-based and event-based vision data to enable high-temporal-resolution object detection and tracking. We leveraged state-of-the-art frame-based object detectors to initialize tracking by detecting and classifying objects in a scene using synchronous image frames, then generated high-temporal-resolution inter-frame tracking using event data. We developed and compared three different approaches for event-based detection and tracking and analyzed their performances at several temporal resolutions. Moreover, we used a simple and low-cost association metric, that is, Euclidean distance, to match object detections across time. 

We evaluated these approaches using our dataset for two traffic scenes, obtained using a static camera with no ego-motion applied. We collect the data using the DAVIS 240c, which combines a frame-based and an event-based sensor using the same lens, generating synchronized image and event data streams. Furthermore, we manually labelled all the vehicles within the scene with accurate bounding boxes and an object ID for every trajectory, using the images generated by the frame-based camera. Then, we generated high-temporal-resolution ground truth trajectories, for object detection and tracking, by temporally interpolating the labeled data, for the tracking rates of 48, 96, 192, and 384 Hz. Finally, we evaluated the results of our different approaches and corresponding configurations using HOTA and a select few CLEAR MOT metrics.

Our results show that out of the three methods presented, event-based masks, combined with temporal weighting of events and a sufficient temporal history, yielded the most consistent performance with minimal deterioration as we progressively increased the tracking rates and the corresponding temporal resolutions, when compared with the baseline frame-based performance at 24 Hz. Moreover, edge-based masks with temporal weighting showed promise as well, ranking very close to the prior approach, whereas our first approach, using event-based masks but without temporal weighting, resulted in the worst performance with the most degradation as we increased the temporal resolutions.

In conclusion, our work shows that a hybrid approach that leverages both image and event data to generate higher tracking temporal resolutions is feasible, with very consistent performance. Our labeled dataset provides a quantitative means of assessing different event-based tracking approaches, which we hope will encourage the production of other challenging labeled event-based datasets for object tracking in the future, given that the presented dataset might not provide the most challenging scenarios that would require more sophisticated detection and tracking approaches. This can be attributed to the relatively low number of available object occlusions and objects present in the scene at any given instant, as well as the limited resolution of the event-based sensor used. Moreover, when considering tracking different object types, we note that classical approaches might not be ideal for objects of dynamic shapes that change at very rapid rates.

This work opens doors for future research, such as into the use of more advanced association metrics tailored for both of these sensing modalities, a more dynamic approach that is less dependent on either, or the exploration of a fully event-based approach for the entire object detection and tracking process.

## Figures and Tables

**Table 1:** Hybrid object detection and tracking results using HOTA metrics at different temporal resolutions, using the frame-based object detector SSD. The results are shown for the three different event-based, inter-frame, tracking modes described in Section 3. Our approaches represented by Modes 2 and 3 show significant promise regarding the ability to leverage event data to generate accurate high-temporal-resolution tracking results. The best metric value at each rate is in bold.

Object Detector	Tracking Rate	Mode	HOTA	DetA	AssA	DetRe	DetPr	AssRe	AssPr	LocA	RHOTA	HOTA(0)	LocA(0)	HOTA-LocA(0)
**SSD**	24 Hz *	-	**69.0**	67.4	70.9	69.7	89.2	73.4	90.1	89.1	70.2	77.2	87.9	67.9
	48 Hz	1	66.6	64.9	68.5	67.0	88.9	70.1	91.1	88.9	67.8	74.9	87.7	65.6
		2	**69.0**	**67.2**	**71.0**	**69.4**	**89.1**	**72.6**	**91.3**	**89.0**	**70.2**	**77.3**	**87.8**	**67.9**
		3	67.6	66.1	69.3	68.2	89.0	70.8	90.9	88.9	68.7	75.9	87.8	66.6
	96 Hz	1	61.0	59.2	63.0	62.1	86.4	64.8	89.6	88.3	62.5	69.4	86.5	60.0
		2	**66.4**	**64.5**	**68.6**	**67.8**	**87.1**	**70.4**	**90.3**	**88.9**	**68.1**	**74.9**	**87.1**	**65.2**
		3	64.4	62.9	66.0	66.0	86.9	67.9	89.8	88.7	66.0	72.9	86.9	63.3
	192 Hz	1	55.0	52.3	58.0	55.0	84.9	59.5	88.6	87.8	56.4	63.0	85.9	54.1
		2	**65.7**	**63.2**	**68.5**	**66.9**	**86.0**	**70.4**	**90.1**	**88.8**	**67.7**	**74.1**	**86.9**	**64.5**
		3	63.3	61.3	65.7	64.8	85.8	67.4	89.7	88.7	65.2	71.7	86.8	62.2
	384 Hz	1	46.3	42.2	50.8	44.0	84.1	52.0	88.0	87.3	47.3	53.2	85.2	45.4
		2	**65.0**	**62.5**	**67.8**	**66.4**	**85.4**	**69.6**	**90.2**	**88.8**	**67.1**	**73.2**	**87.0**	**63.7**
		3	62.5	60.4	64.7	64.2	85.2	66.4	89.8	88.7	64.4	70.6	86.9	61.3

* Image frames only.

**Table 2:** Hybrid object detection and tracking results using HOTA metrics at different temporal resolutions, using the frame-based object detector YOLOv3. The results are shown for the three different event-based, inter-frame, tracking modes described in Section 3. Our approaches represented by modes 2 and 3 show significant promise regarding the ability to leverage event data to generate accurate high-temporal-resolution tracking results. The best metric value at each rate is in bold.

Object Detector	Tracking Rate	Mode	HOTA	DetA	AssA	DetRe	DetPr	AssRe	AssPr	LocA	RHOTA	HOTA(0)	LocA(0)	HOTA-LocA(0)
**YOLOv3**	24 Hz *	-	**56.6**	53.0	60.8	54.6	83.6	62.9	87.0	84.2	57.5	68.1	82.0	55.9
	48 Hz	1	53.8	51.2	56.8	52.7	83.5	58.8	86.7	84.1	54.6	65.0	81.9	53.2
		2	**55.4**	**52.9**	**58.4**	**54.5**	83.6	**60.4**	**86.9**	84.2	**56.4**	**66.9**	82.0	**54.9**
		3	54.7	52.3	57.5	53.8	**83.7**	59.5	86.4	**84.3**	55.6	66.0	**82.1**	54.2
	96 Hz	1	49.6	47.1	52.4	49.0	81.6	53.8	86.4	83.8	50.6	60.6	80.9	49.1
		2	**53.6**	**51.4**	**56.2**	**53.5**	82.1	**57.7**	**86.9**	84.1	**54.8**	**65.3**	81.3	**53.1**
		3	52.6	50.5	55.1	52.4	**82.2**	56.6	86.3	**84.2**	53.7	64.1	**81.4**	52.2
	192 Hz	1	44.4	41.6	47.6	43.3	80.9	48.9	85.8	83.8	45.4	54.1	80.9	43.7
		2	**53.2**	**50.5**	**56.2**	**52.9**	**81.2**	**57.7**	**86.7**	84.1	**54.5**	**64.8**	81.2	**52.6**
		3	52.0	49.4	54.9	51.6	81.4	56.4	86.1	**84.2**	53.2	63.3	**81.4**	51.5
	384 Hz	1	36.4	33.1	40.2	34.2	80.9	41.1	85.3	83.8	37.0	44.0	81.0	35.6
		2	**52.5**	**50.1**	**55.3**	**52.6**	80.8	**56.7**	**87.0**	84.1	**53.9**	**63.8**	81.3	**51.9**
		3	51.3	48.9	54.1	51.2	**80.9**	55.5	86.2	**84.2**	52.6	62.3	**81.5**	50.8

* Image frames only.

**Table 3:** Hybrid object detection and tracking results using a subset of CLEAR MOT metrics for different tracking configurations and temporal resolutions. The selected metrics provide extra insight into the behavior and the quality of each tracking configuration. Mode 2′s tracking configuration consistently outperformed the others in all metrics, deteriorating slightly with increasing temporal resolutions. The best metric value at each rate is in bold.

Tracking Rate	Mode	MT	PT	ML	IDSW	FRAG	MT	PT	ML	IDSW	FRAG
		SSD					YOLOv3				
24 Hz *	-	**40**	45	0	16	**33**	**27**	52	6	18	21
48 Hz	1	39	46	0	29	70	21	58	6	30	76
	2	**40**	45	0	29	**34**	**27**	52	6	30	**22**
	3	**40**	45	0	29	35	25	54	6	30	23
96 Hz	1	25	59	1	48	749	15	62	8	50	550
	2	**37**	47	1	48	**715**	**25**	53	**7**	50	**502**
	3	36	48	1	48	727	23	55	**7**	50	517
192 Hz	1	16	65	4	49	908	8	66	11	54	635
	2	**37**	46	**2**	**48**	**715**	**25**	52	**8**	**50**	**505**
	3	35	48	**2**	**48**	738	23	54	**8**	**50**	526
384 Hz	1	9	68	8	67	1054	3	69	13	86	695
	2	**37**	46	**2**	**61**	**721**	**25**	52	**8**	**75**	**507**
	3	35	48	**2**	62	742	22	55	**8**	**75**	532

* Image frames only.

## Data Availability

The data presented in this study are openly available at http://sar-lab.net/event-based-vehicle-detection-and-tracking-dataset/ (accessed on 22 July 2022).

## Appendix A

**Table A1:** The total number of IDs and detections for both the ground truth data and the predicted results for the different tracking configurations used in our evaluation. Our dataset has a total number of unique object ID trajectories of 85. The number of ground truth detections increases as the temporal resolution of the data increases.

		Ground Truth		SSD		YOLOv3	
Tracking Rate	Mode	GT_IDs	GT_Dets	IDs	Dets	IDs	Dets
24 Hz *	-	85	9891	110	7723	105	6462
48 Hz	1	85	19,777	125	14,908	117	12,480
	2			125	15,405	117	12,899
	3			125	15,155	117	12,710
96 Hz	1	85	39,549	147	28,427	139	23,748
	2			147	30,773	139	25,773
	3			147	30,022	139	25,207
192 Hz	1	85	79,093	149	51,226	142	42,332
	2			147	61,498	139	51,521
	3			147	59,720	139	50,179
384 Hz	1	85	158,181	171	82,708	172	66,862
	2			165	122,972	165	103,023
	3			165	119,131	165	100,131

* Image frames only.
